# The novel RAGE interactor PRAK is associated with autophagy signaling in Alzheimer’s disease pathogenesis

**DOI:** 10.1186/s13024-016-0068-5

**Published:** 2016-01-12

**Authors:** Yoonhee Kim, Chaeyoung Kim, Sung Min Son, Hyundong Song, Hyun Seok Hong, Sun-ho Han, Inhee Mook-Jung

**Affiliations:** Department of Biochemistry and Biomedical Sciences, Seoul National University College of Medicine, 103 Daehak-ro, Jongro-gu, Seoul 110-799 Korea

**Keywords:** PRAK, RAGE, Alzheimer’s disease, Autophagy, Aβ

## Abstract

**Background:**

The receptor for advanced glycation end products (RAGE) has been found to interact with amyloid β (Aβ). Although RAGE does not have any kinase motifs in its cytosolic domain, the interaction between RAGE and Aβ triggers multiple cellular signaling involved in Alzheimer’s disease (AD). However, the mechanism of signal transduction by RAGE remains still unknown. Therefore, identifying binding proteins of RAGE may provide novel therapeutic targets for AD.

**Results:**

In this study, we identified p38-regulated/activated protein kinase (PRAK) as a novel RAGE interacting molecule. To investigate the effect of Aβ on PRAK mediated RAGE signaling pathway, we treated SH-SY5Y cells with monomeric form of Aβ. We demonstrated that Aβ significantly increased the phosphorylation of PRAK as well as the interaction between PRAK and RAGE. We showed that knockdown of PRAK rescued mTORC1 inactivation induced by Aβ treatment and decreased the formation of Aβ-induced autophagosome.

**Conclusions:**

We provide evidence that PRAK plays a critical role in AD pathology as a key interactor of RAGE. Thus, our data suggest that PRAK might be a potential therapeutic target of AD involved in RAGE-mediated cell signaling induced by Aβ.

**Electronic supplementary material:**

The online version of this article (doi:10.1186/s13024-016-0068-5) contains supplementary material, which is available to authorized users.

## Background

Alzheimer’s disease (AD), a progressive neurodegenerative disorder, is the most common type of dementia [1]. Notably, Amyloid β (Aβ) is a major pathological characteristic of AD [2]. Along with neurofibrillary tangles and neuronal loss, Aβ influences AD pathogenesis, including oxidative injury, synaptic degeneration, inflammatory response and neuronal death. Unfortunately, the intermediate mechanism underlying toxic Aβ interactions and AD pathogenesis remains unelucidated. As a result, current treatments can merely alleviate AD symptoms and delay deterioration [3].

The receptor for advanced glycation end-products (RAGE) is a multi-ligand receptor that belongs to the immunoglobulin superfamily [4, 5]. RAGE ligands are comprised of advanced glycation end-products [4], high mobility group box 1 (HMGB1, also known as amphoterin) [6], S100/calgranulins [7, 8], Mac-1 [9], phosphatidylserine [10] and Aβ [11]. Interactions of ligand-RAGE activate multiple intracellular signaling pathways involving MAPKs such as ERK1/2, p38, JNK, PI3K, Src kinase, JAK/STAT, TGFβ/Smad, and members of the Rho GTPase signaling pathway [12]. Moreover, ligand-RAGE interactions cause the generation of reactive oxygen species [13], influence cellular homeostasis and inflammatory response, and lead to diseases such as cancer, diabetes and AD [14].

Multiple lines of evidence underscore the importance of the 42 amino acids of the RAGE cytoplasmic domain in intracellular signal transduction. For example, deletion in this RAGE cytoplasmic domain (DN-RAGE) renders RAGE incapable of facilitating signal transduction following ligand-RAGE interaction [15]. Furthermore, the absence of any known signal transduction motifs in the RAGE cytoplasmic domain has limited our understanding of AD pathogenesis through Aβ-RAGE interaction-mediated signaling. mDia-1 is known to interact with the RAGE cytoplasmic domain and requires RAGE-mediated cellular migration, Rho GTPases (particularly cdc42 and rac-1) activation for this interaction [16]. However, RAGE cytoplasmic domain binding proteins responsible for triggering other RAGE-mediated signaling pathways remain unknown.

p38-regulated/activated protein kinase (PRAK), also known as the mitogen-activated protein kinase (MAPK) activated protein kinase-5 (MK5), is a Ser/Thr protein kinase and a member of the MAPKs [17]. PRAK can be activated by cellular stress and inflammatory cytokines [18]. PRAK is known to phosphorylate Heat shock protein 27 (HSP27) [17], cytosolic phospholipase A2 (cPLA2) [19], tyrosine hydroxylase [20], FOXO3a [21], FAK [22], septin8 [23] and p53 [24]. Thus, PRAK plays a crucial role in cellular signaling phenomena such as the cell cycle, angiogenesis, and neuronal plasticity [25]. Moreover, PRAK regulates the phosphorylation of Ras homologue enriched in brain (Rheb), a main component of mammalian target of rapamycin complex 1 (mTORC1), leading to decreased cell growth [26].

In this study, we found PRAK as a novel interactor of the RAGE cytoplasmic domain using the yeast two-hybrid approach. The interaction between PRAK and RAGE was further verified by immunoprecipitation (IP), surface plasmon resonance (SPR). We identified that Aβ treatment induced phosphorylation of PRAK and increased interaction between PRAK and RAGE. More interestingly, the interaction between PRAK and RAGE was also increased in the brains of Tg6799 mouse, AD animal model. Furthermore, knockdown of PRAK reduced RAGE-mediated formation of autophagosome via mTORC1 signaling pathway. Our results indicate that PRAK binds to the RAGE cytoplasmic domain and regulates Aβ-RAGE-mediated autophagy.

## Results

### Identification of PRAK as a binding partner of RAGE

**Fig. 1:**
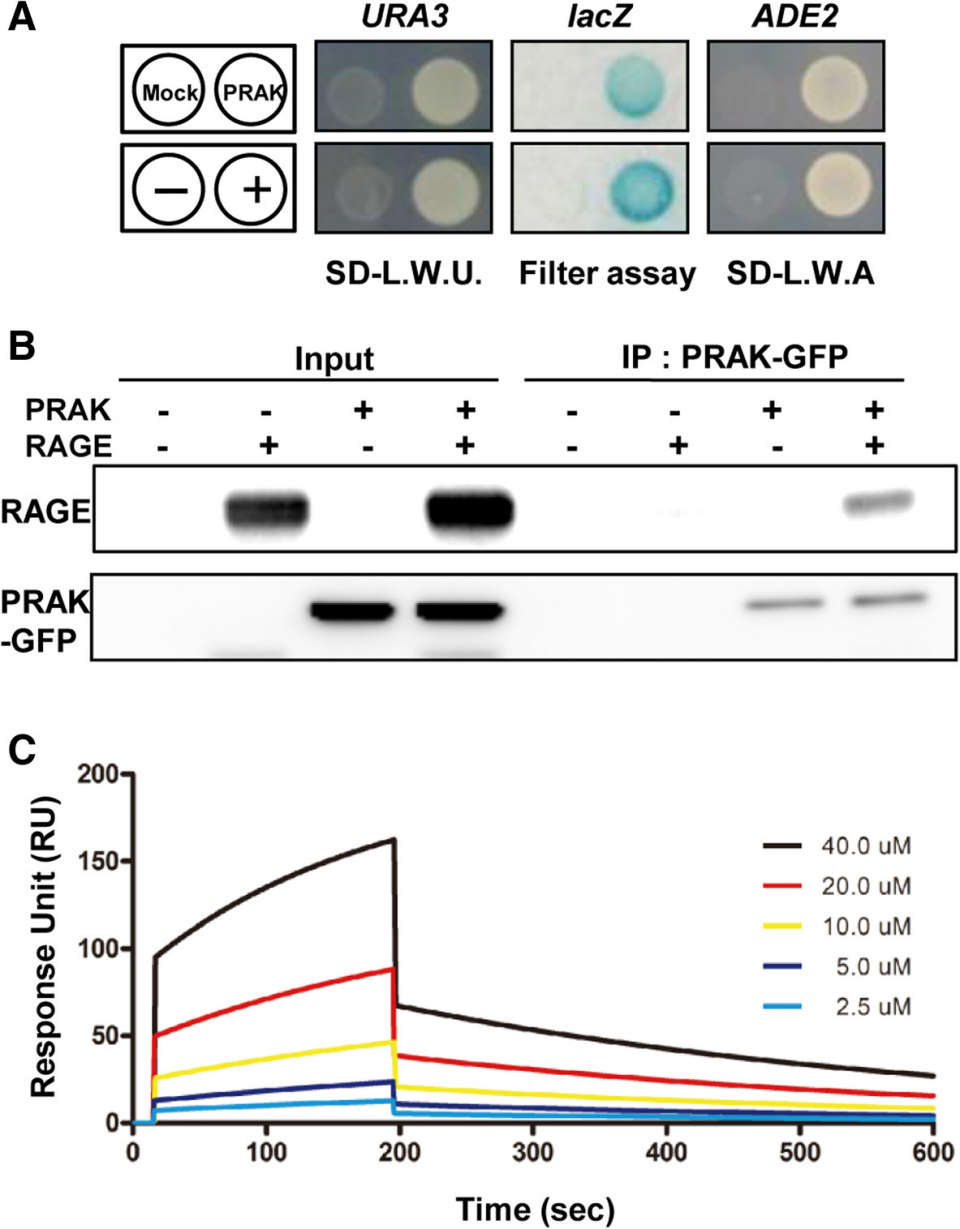
PRAK interacts with the RAGE. **a** Yeast two-hybrid screening to identify RAGE interacting proteins in a human brain cDNA library. Yeast transformants of the RAGE bait and human brain cDNA library were spread on selection medium SD-LWU (SD without leucine, tryptophan and uracil), SD-LWA (SD without leucine, tryptophan and adenosine) and filter assay. pGBKT- PTB and pACT2-PTB served as the positive control (+). pGBKT and pACT2 were used as negative control (−). **b** PRAK and RAGE binding in vitro. Immunoprecipitation (IP) with anti-GFP antibody was accomplished using lysates from CHO cells transfected with RAGE and GFP-tagged PRAK, followed by western blotting with anti-RAGE antibody. **c** Binding kinetics of the RAGE C-term to PRAK protein. GST-fused PRAK protein was immobilized onto a CM5 sensor chip as the ligand. The RAGE C-term was used as the analyte from 0 to 40 μM to measure the kinetics of binding. Curves corresponding to multiple analyte concentrations were generated to ensure the precision of the calculation of the kinetics. The binding kinetics were analyzed using BIAevaluation 3.1 software

To identify proteins that interact with the RAGE cytoplasmic domain, we carried out the yeast two-hybrid screening between the human brain cDNA library and RAGE cytoplasmic domain, the latter was used as the bait. We identified interaction between PRAK and the RAGE cytoplasmic domain through the expression of three reporter genes, *lacZ*, *ura3*, and *ade2*, each being under the control of different GAL4 promoters. To ensure the reliability of the results, both the positive and negative controls were incorporated on the same filter (Fig. 1a). To validate the interaction between PRAK and RAGE, we transfected PRAK-GFP and RAGE into cells and performed immunoprecipitation with anti-GFP antibodies (Fig. 1b). We also confirmed the interaction between PRAK and RAGE at endogenous levels in SH-SY5Y cells by antibodies against both PRAK and RAGE (Additional file 1: Figure S1). We investigated the binding kinetics between PRAK and RAGE using surface plasmon resonance (SPR) by immobilizing PRAK protein on the chip surface. The response unit (RU) was gradually increased by the RAGE concentration from 2.5 μM to 40 μM (Fig. 1c). The K_d_ value of the binding strength of the RAGE cytoplasmic domain to PRAK is 0.5086 nM. This SPR data supported that the PRAK is a specific binding protein to the RAGE cytosolic domain.

### Aβ increases PRAK-RAGE interaction

**Fig. 2:**
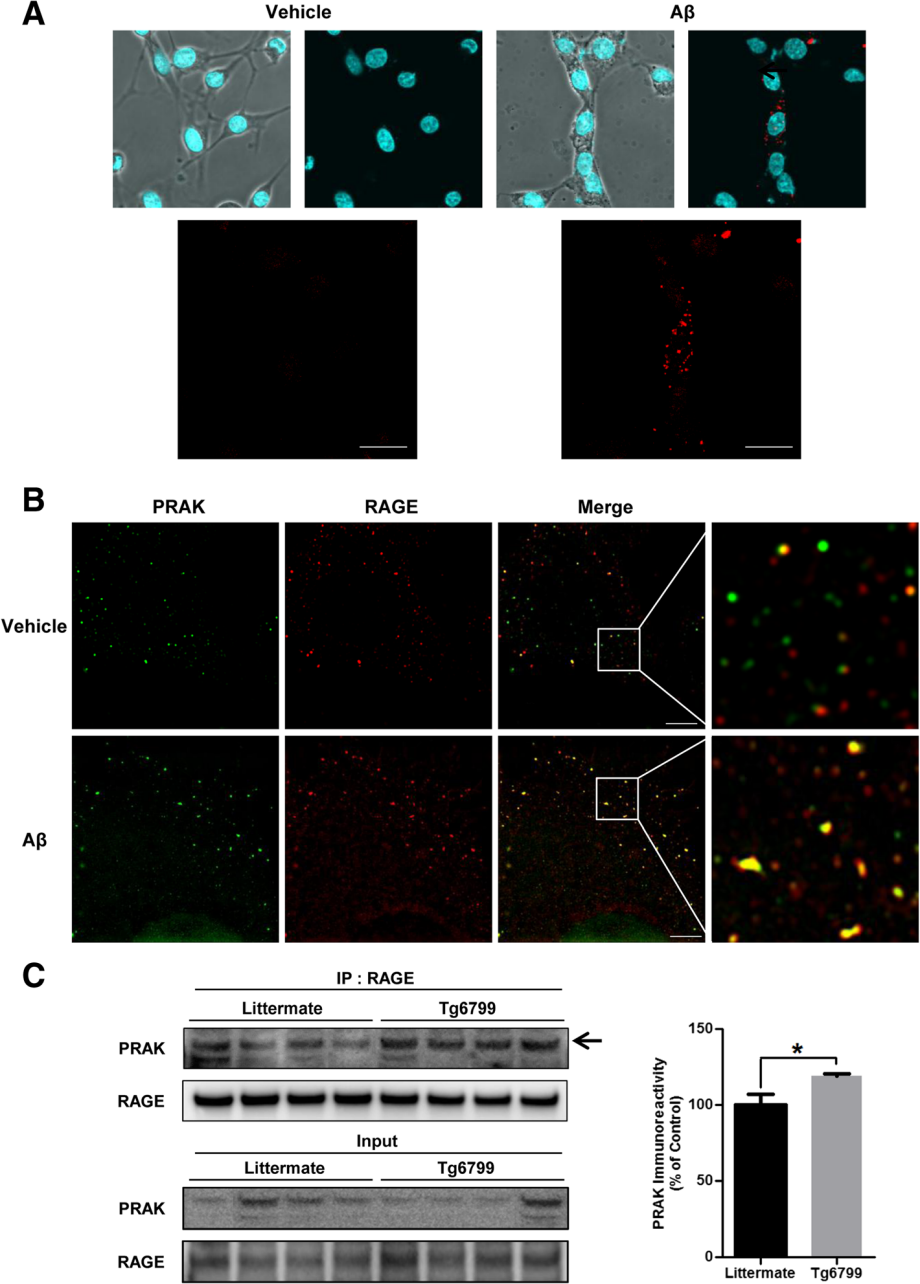
Aβ treatment increases the interaction between PRAK and RAGE. **a** Aβ treatment increases RAGE-PRAK binding. Overexpression of RAGE and PRAK in SH-SY5Y cells were treated with either DMSO or monomeric Aβ 2 μM for 6 h and co-localization was detected using in situ proximity ligation assay kit. Probes in close proximity (<40 nm) are indicated by fluorescent dot signals. Scale bars represent 20 μm. **b** Aβ enhances the co-localization between RAGE and PRAK. SH-SY5Y cells was induced by a 6 h treatment of DMSO or monomeric 2 μM Aβ. Immunofluorescence analysis using anti-PRAK (green) and anti-RAGE (red) antibodies. SIM was used for image analysis. Scale bars represent 5 μm. **c** PRAK and RAGE binding in vivo. Immunoprecipitation (IP) with anti-RAGE antibody was accomplished using total brain lysates from 3 months wild-type litter mates and Tg6799, followed by western blotting with anti-PRAK antibody. Quantification of protein level was performed by densitometric analysis. Data are mean ± SEM. n = 4. *P < 0.05

The binding of RAGE to its ligands triggers various signaling pathways and this process is dependent on the RAGE cytoplasmic domain. In AD, RAGE expression is increased in the brain [11]. Aβ-RAGE interaction induces the cellular effects associated with AD pathology [27–29]. To investigate the effect of Aβ on interaction between PRAK and RAGE, we performed in situ proximity ligation assay on cells overexpressing both RAGE and PRAK after 2 μM monomeric Aβ treatment. The red dots suggest closely apposed binding of the two proteins (~40 nm). The red dot signals increased substantially in treatment of cells with Aβ compared to treatment of cell with vehicle (Fig. 2a). Furthermore, to see the colocalization between PRAK and RAGE, we used the structure illumination microscopy (SIM) with high resolution. After treatment of monomeric Aβ for 6 h, we stained using anti-PRAK antibody (green) and anti-RAGE antibodies (red). Compared to vehicle treatment, Aβ treatment increased the colocalization between PRAK and RAGE (Fig. 2b). These data indicate that Aβ treatment increases the interaction between PRAK and RAGE. In addition, we confirmed the interaction between PRAK and RAGE in vivo using the brains of Tg6799 mice as AD animal model. Consistent with cell data using Aβ, the binding of PRAK to RAGE was increased in the brains of Tg6799 compared to litter mate (Fig. 2c, 19.2 % ± 1.684, * *p* < 0.05, n = 4 independent experiments). Taken together, these data implicate that the interaction between PRAK and RAGE was increased under Aβ abundant conditions in cells as well as in vivo.

### Aβ induces phosphorylation of PRAK via RAGE-intracellular domain

**Fig. 3:**
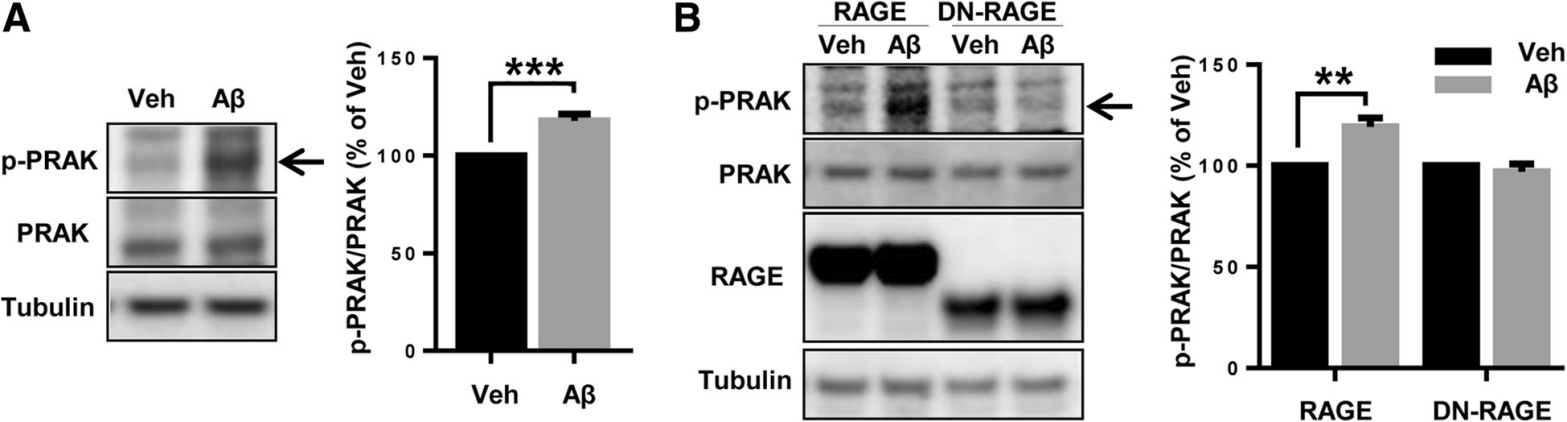
Aβ treatment induces phosphorylation of PRAK via RAGE. **a** SH-SY5Y cells were treated with either DMSO or 2 μM monomeric Aβ for 6 h. **b** SH-SY5Y cells were transfected with full length RAGE and DN-RAGE and subsequently treated with either DMSO or 2 μM monomeric Aβ for 6 h. Western blot was performed with the indicated antibodies. Quantification of protein level was performed by densitometric analysis. Data are mean ± SEM of at least 3 independent experiments. **P < 0.01, ***P < 0.001

Since PRAK is a protein kinase activated through phosphorylation in response to cellular stress and proinflammatory cytokine [17]. We examined whether 2 μM monomeric Aβ treatment can induce phosphorylation of PRAK. As expected, Aβ treatment increased the level of phosphorylated PRAK at Thr-142 compared to vehicle. The mean change was about 18 % ± 3.3 (*** *p* < 0.001, n = 4 independent experiments, Fig. 3a). To test whether the RAGE cytoplasmic domain, the PRAK binding site, is critical for phosphorylation of PRAK after Aβ stimulation, we used cells overexpressing RAGE or the RAGE cytoplasmic domain deletion mutant (DN-RAGE). Full length of RAGE induced PRAK phosphorylation by Aβ stimulation compared with the vehicle (19.4 % ± 4.38, ** *p* < 0.01, n = 3 independent experiments). However, phosphorylation of PRAK was not altered by Aβ treatment in cells overexpressing DN-RAGE (Fig. 3b). This result suggests that phosphorylation of PRAK induced by Aβ treatment is dependent on RAGE-cytoplasmic domain.

### Aβ induces mTORC1 inactivation via PRAK and RAGE

**Fig. 4:**
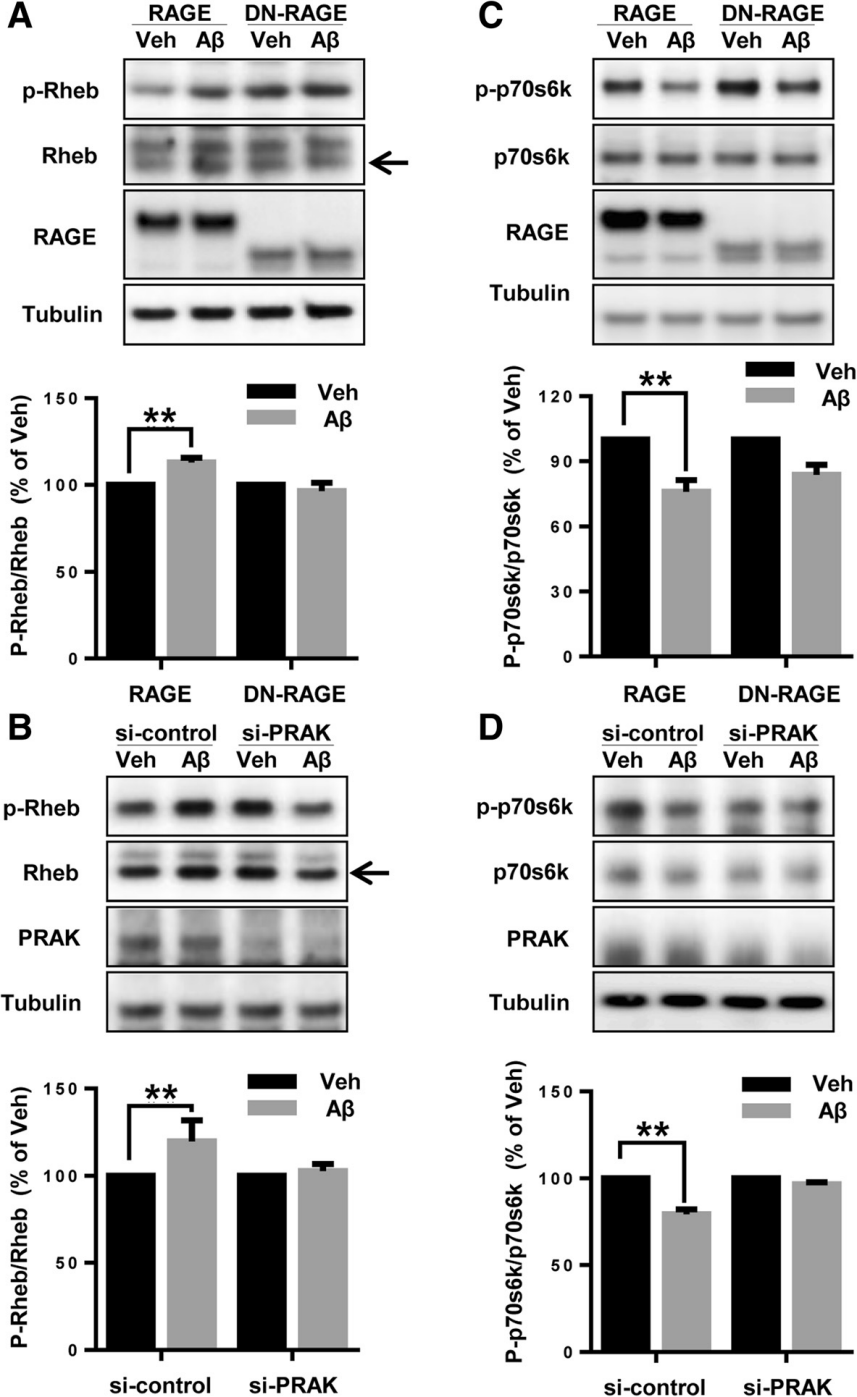
RAGE and PRAK mediate mTORC1 inactivation by Aβ treatment. **a** RAGE and DN-RAGE-overexpressing SH-SY5Y cells were treated with either DMSO or 2 μM monomeric Aβ for 6 h. **b** SH-SY5Y cells were transfected with plasmids expressing RAGE and then transiently re-transfected with PRAK siRNA or scrambled control siRNA. Cells were treated with either DMSO or 2 μM monomeric Aβ for 6 h. **c** RAGE and DN-RAGE-overexpressing SH-SY5Y cells were treated with either DMSO or 2 μM monomeric Aβ for 6 h. **d** SH-SY5Y cells were transfected with plasmids expressing RAGE and then transiently re-transfected with PRAK siRNA or scrambled control siRNA. Cells were treated with either DMSO or 2 μM monomeric Aβ for 6 h. Western blot was performed with the indicated antibodies. Quantification of protein level was performed by densitometric analysis. Data are mean ± SEM of 4 separate experiments. **P < 0.01

mTORC1/p70S6K signaling associated with autophagy formation is one of Aβ induced downstream signaling pathways [29]. In addition, Rheb, the main component of the mTORC1 complex, is a downstream substrate of PRAK [26]. We investigated the status of Rheb and mTORC1/p70S6K activation as a downstream signaling pathway of Aβ induced PRAK-RAGE interaction. To see if alteration of phosphorylation on Rheb or mTORC1/p70S6k induced by Aβ is dependent on both PRAK and RAGE, we used SH-SY5Y cells overexpressing DN-RAGE or siPRAK. 2 μM monomeric Aβ treatment increased phosphorylation of Rheb, compared with the vehicle (13.1 % ± 2.55, ** *p* < 0.01, n = 4 independent experiments, Fig. 4a in RAGE overexpressed cells). In contrast, Aβ treatment did not alter phosphorylation of Rheb in cells overexpressing DN-RAGE (n = 4 independent experiments, Fig. 4a). Consistent with the results of Fig. 4a, knockdown of PRAK using siRNA against PRAK did not significantly increase phosphorylation of Rheb even in the presence of Aβ (in the case of control si-RNA treated group: 19.8 % ±9.06 increase in Aβ treated cells compared to vehicle treated cells, ** *p* < 0.01, n = 4 independent experiments, Fig. 4b). Since phosphorylation of Rheb inhibits the mTORC1/p70S6K pathway [30], we examined the p70s6k phosphorylation levels. Compared with vehicle treatment, phosphorylation of p70s6k was significantly decreased by Aβ treatment (24.1 % ± 5.34, ** *p* < 0.01, n = 4 independent experiments, Fig. 4c). However, Aβ treatment did not significantly decrease p-p70s6k in cells overexpressing DN-RAGE (Fig. 4c). Moreover, deletion of PRAK by si-PRAK did not alter p-p70s6k by Aβ treatment compared with vehicle treated group, while si-control cells decreased p-p70s6k by Aβ treatment compared with vehicle treated group (21.7 % ±2.95, ** *p* < 0.01, n = 4 independent experiments, Fig. 4d). Mock-transfected cells showed similar trends on phosphorylation of Rheb and p70s6k as cells overexpressing RAGE due to endogenous RAGE effects when Aβ was treated even though the degree of phosphorylation is much less (data not shown). Therefore, these results demonstrate that the activation of Rheb-mTORC1/p70S6K induced by Aβ is dependent on PRAK-RAGE interaction.

### PRAK mediates RAGE-Aβ-driven autophagosome formation

**Fig. 5:**
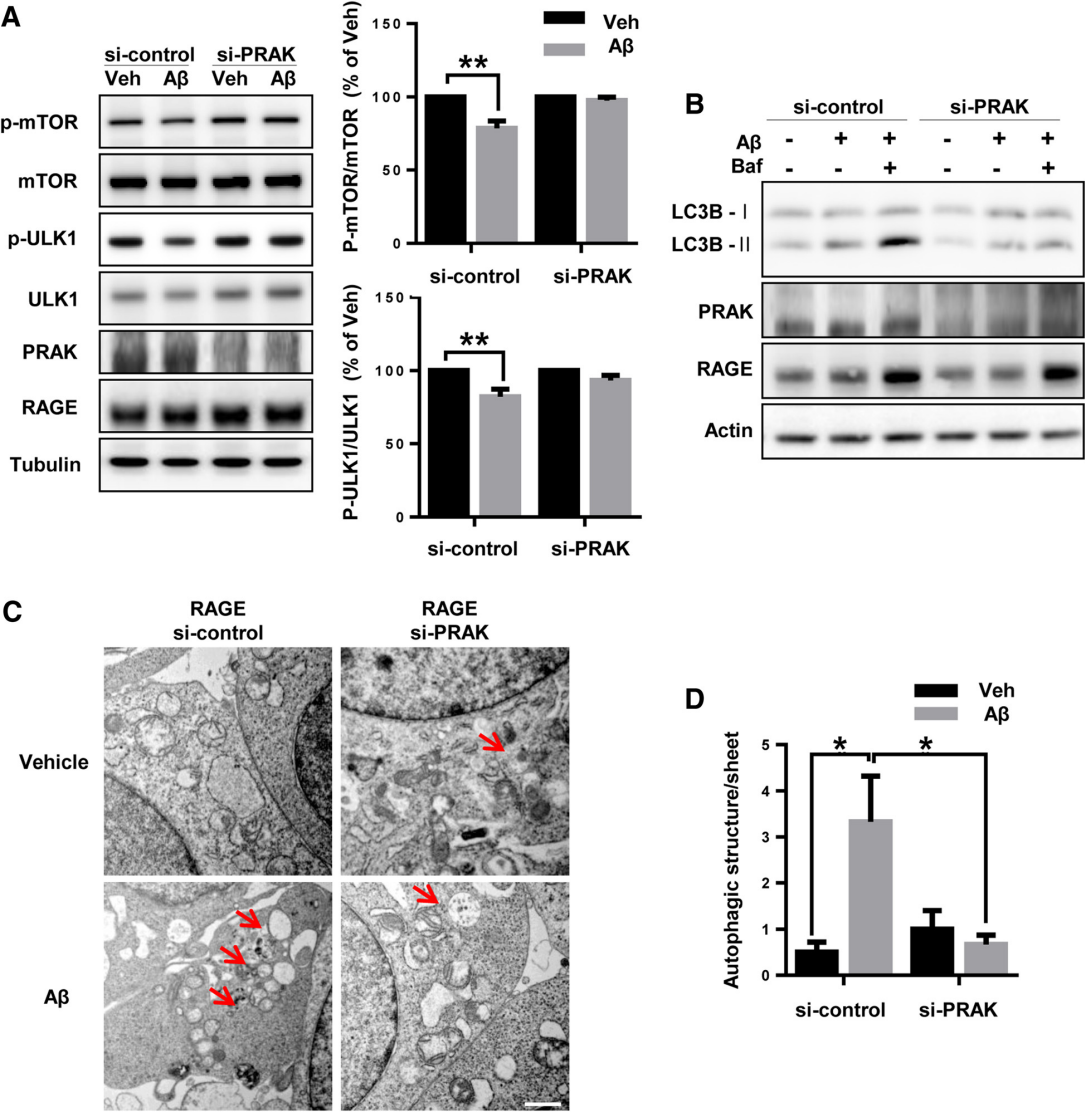
PRAK mediates Aβ-induced autophagy signaling pathway. **a** SH-SY5Y cells were transfected with plasmids expressing RAGE and then transiently re-transfected with PRAK siRNA or scrambled control siRNA. After 24 h, cells were treated with either DMSO or 2 μM monomeric Aβ for 6 h. **b** SH-SY5Y cells were transfected with plasmids expressing RAGE and transiently retransfected with PRAK siRNA or scrambled control siRNA. After 24 h, cells were pretreatment with 10 nM Bafilomycin A_1_ for 1 h and treated with either DMSO or 2 μM monomeric Aβ for 6 h. Western blot analysis was performed with the indicated antibodies. Quantification of protein level was performed by densitometric analysis. Data are mean ± SEM of 5 separate experiments. **P < 0.01. **c** Cells were co-transfected with RAGE plasmid DNA and PRAK siRNA or scrambled control siRNA. After 24 h, cells were treated with either DMSO or 2 μM monomeric Aβ for 6 h, fixed, and stained for TEM as described in the methods section. EM images indicate PRAK-mediated changes in the number of autophagosomes (arrows). Quantification of autophagosomes is shown in (**d**). Scale bars represent 1 μm. *P < 0.05

Since autophagosome formation is one of the Rheb-mTOR downstream effects, we further examined whether PRAK involves formation of autophagosome induced by Aβ. Since activation of mTOR suppresses autophagy induction by phosphorylation of serine/threonine kinases, UNC-51-like kinase 1 (ULK1) [31], we measured phosphorylation of mTOR and ULK1 as an early marker of autophagy induction. ULK1, a mammalian autophagy initiating kinase, plays a critical role in the early stage of autophagy [31]. 2 μM monomeric Aβ treatment significantly decreased the phosphorylation of mTOR and ULK1 compared with vehicle (mTOR: 21.4 % ±4.96, ULK1: 17.8 % ±5.21 ** *p* < 0.01, n = 5 independent experiments). However, the levels of p-mTOR and p-ULK were not changed by Aβ treatment in cells transfected with siRNA against PRAK (Fig 5a). Because microtubule-associated protein 1A/1B-light chain 3-II (LC3-II) is increased during formation of autophagosome [32] and Aβ is known to induce autophagosome formation [33], we measured the level of LC3-II by Western blot. 2 μM monomeric Aβ treatment induced accumulation of LC3-II, but siRNA against PRAK transfected cells reduced accumulation of LC3-II by Aβ treatment compared to vehicle (Fig. 5b). The accumulation of LC3-II could be due to either formation of autophagosome or blockage of downstream in autophagy (autophagic flux) [32]. To distinguish these two possibilities, we used bafilomycin A_1_ that blocks fusion of autophagosome with lysosomes to make autolysosome, resulting in accumulation of LC3-II. Bafilomycin A_1_ with Aβ treatment increased accumulation of LC3-II compared with only Aβ treatment (Fig. 5b, si-control lanes). In contrast, knockdown of PRAK by siRNA against PRAK did not induce accumulation of LC3-II level by Bafilomycin A_1_ with Aβ treatment compared with only Aβ treatment (Fig. 5b, si-PRAK lanes). Taken together, it implies that PRAK mediated accumulation of LC3-II is due to autophagy formation, not by blockage of autophagic flux. To evaluate actual autophagosome formation via PRAK, we used transmission electron microscopy (TEM) to examine the cellular ultrastructure. As shown in Fig. 5c and d, Aβ treatment increased the accumulation of autophagosomes (red arrows), compared with vehicle. In contrast, deletion of PRAK did not induce accumulation of autophagosomes by Aβ treatment. Taken together, these results demonstrate that the Aβ-RAGE-induced autophagosome formation is mediated by PRAK.

## Discussion

RAGE mediates diverse cellular signaling pathways triggered by specific ligands [34]. In this study, we provide evidence that alteration of Rheb-mTOR/p70S6K by interaction of Aβ-RAGE is mediated by PRAK. Since Aβ-RAGE interaction also leads to the activation of an NF-κB dependent signaling pathway [11], we investigated if PRAK can modulate activation of NF-κB induced by Aβ-RAGE interaction. As expected, Aβ treatment increased p-IκB compared with vehicle treatment. However, knockdown of PRAK did not change the level of p-IκB by Aβ treatment (Additional file 2: Figure S2). Therefore, our data support that PRAK specifically mediates Rheb-mTORC1/p70S6K signaling pathway among various downstream signaling pathways of Aβ-RAGE interaction.

Although ligands of RAGE and their associated signaling pathways are well known, there are few studies of RAGE cytoplasmic domain-interacting proteins [28]. In this study, we identified that PRAK as an interactor of the RAGE is recruited and phosphorylated by Aβ treatment and interact with RAGE cytoplasmic domain. Previous studies reported that PRAK, a substrate for atypical and conventional MAPKs, is phosphorylated and activated by p38 and ERK3/4 [17, 35]. In addition, it is well known that p38 is phosphorylated by Aβ mediated-RAGE activation [12, 36]. To investigate if p38 phosphorylates PRAK by Aβ-RAGE interaction, we measured the level of phosphorylated p38 using western blot. Consistent with previous studies, the interaction between Aβ-RAGE activates p38 (Additional file 3: Figure S3). However, p38 is also phosphorylated by Aβ treatment in DN-RAGE overexpressing cells. This data suggests that PRAK might be phosphorylated by the other MAPK, but not p38, because PRAK is not phosphorylated by Aβ treatment in DN-RAGE overexpressing cell (Fig 3b).

Zheng et al., reported that PRAK regulates mTORC1 by phosphorylation of Rheb. They also showed that alteration of mTORC1 by PRAK is independent of AMP-activated protein kinase (AMPK) and Tuberous Sclerosis Complex 2 (TSC2) [26]. Our data also suggest that interaction between RAGE and PRAK by Aβ treatment regulate mTORC1, leading to autophagosome formation. The mTOR forms two catalytic distinct complexes: mTOR complex 1 (mTORC1) and mTOR complex 2 (mTORC2). mTORC1 and mTORC2 are regulated by different upstream and downstream components. mTORC1 is regulated by various upstream effects, such as PI3K/Akt, GSK-3β, AMPK, LKB1, IRS-1 and MAPK [37–41]. There are a number of downstream components of mTORC1, including STAT3, 4EBPs and p70S6Ks [42]. mTORC1 is highly expressed in brain and regulates multiple signaling pathways related with protein synthesis, autophagy, cell growth and mitochondrial function [43]. Accumulating evidences suggest that mTORC1 plays a critical role in AD pathology [44, 45]. In a previous study, we demonstrated that interaction between Aβ and RAGE induces autophagic vacuoles via the Ca^2+^/calmodulin-dependent protein kinase kinase-AMPK pathway [29]. In this study, we focused our efforts on autophagy among several PRAK downstream signals and found that Aβ treatment increases the interaction between RAGE and PRAK, inducing autophagosome formation via Rheb-mTORC1/p70s6k. Therefore, we further provided evidence that Aβ-RAGE-mediated autophagosome formation is regulated not only by CaMKKβ-AMPK signaling through calcium, but also by Rheb-mTORC1 signaling through PRAK. Inhibition of mTOR can induce autophagosome formation [46] and many autophagic vacuoles can be seen in the brains of AD patients [33, 47]. When beclin 1, one of autophagy initiating molecules, was knocked down in neurons and transgenic mice in AD mouse model, less autophagosome were shown and more Aβ depositions were examined [48, 49], suggesting autophagy has a beneficial role to remove Aβ accumulation. However, since the disruption of lysosomal function has been reported in the brains of AD patients and animal models [50, 51], autophagic flux, fusion between autophagosome and lysosome, might be impaired, resulting in less autolysosomes. It causes the inhibition of abnormal protein degradation [52, 53]. Many autophagic vacuoles in the brains of AD patients might be resulted in accumulation of autophagosomes due to blockade of autophagic flux with lysosomal dysfunction. Out data showed that Aβ-RAGE-PRAK axis stimulates autophagic formation via inhibiting mTORC1/p70s6k to make autophagosome. If the function of lysosome is intact in the brains, Aβ-RAGE-PRAK axis-induced autophagosomes might be beneficial. Since the exact status of lysosomes in each stages of AD is still unknown, the role of autophagy in AD pathogenesis is controversial. The detailed mechanism regarding autophagic flux in AD should be clarified as a further study.

## Conclusions

In summary, we identified that PRAK is a novel RAGE interactor, and mediates downstream signaling by direct binding to the cytoplasmic domain of RAGE. Specifically, PRAK mediates the Rheb-mTORC1/p70s6k pathway by Aβ-RAGE interaction. Our data suggest that PRAK is a critical regulating factor that modulates RAGE downstream signaling and RAGE-induced AD pathology.

## Methods

### Yeast two-hybrid screening

Yeast two-hybrid screening was performed by Panbionet, (http://www.panbionet.com/), with the RAGE carboxyl terminal (C-terminal) region (BD, binding domain) and the human brain cDNA domain (AD, activation domain) library. RAGE C-terminal region (361-end amino acids of RAGE) was amplified by PCR using the following primers pair: F primer, 5′-GGC GAA TTC ATC TTG TGG CAA AGG CGG–3′; R primer, 5′-GGC AGA TCT TCA AGG CCC TCC AGT ACT–3′. The RAGE bait (135 nt) was cloned into the BamHI/EcoRI sites of the pGBKT vector, which contain the GAL4 DNA binding domain (GAL4DB). The AD library inserts were cloned into pACT2 vector containing a GAL4 activation domain. These two-hybrid plasmids were co-transformed into yeast strain PBN204, containing 3 reporters (*URA3*, *lacZ*, *and ADE2*) under the control of dissimilar GAL4 promoters. In order to confirm the interaction, positive clones were amplified by *Escherichia coli* transformation or PCR. The amplified clones were reintroduced into yeast PBN204 strain with the RAGE bait plasmid or with a negative control plasmid expressing GAL4-binding domain without the bait. pACT2-polypyrimidine tract-binding protein (PTB) and pGBKT-PTB served were used as positive controls for the protein-protein interactions. pACT2 and pGBKT served were used as the negative controls. The identity of the interactors was determined by sequencing.

### Cell culture and animals

SH-SY5Y human neuroblastoma and Chinese hamster ovary (CHO) cell lines were maintained in Dulbecco’s modified Eagle’s medium (DMEM, HyClone) then added to 10 % FBS (HyClone), 0.1 mg/mL penicillin and streptomycin (Sigma) at 37 °C in a 5 % CO_2_ incubator. Tg6799 (B6SJL-Tg [APPSwFlLon, PSEN*M146L*L286V] 6799Vas/J, Jackson Lab, stock no. 006554) and B6SJL wild-type (littermate) mice were used for the experiments. All animal use was performed according to the Principles of Laboratory Animal Care (NIH publication no. 85–23, revised 1985) and use guidelines of Seoul National University, Seoul, Korea.

### Transfection

Approximately 100 × 10^3^ cells were seeded in tissue culture plates and plated at 70 % confluence after 24 h. Cells were transfected with full-length human RAGE or DN-RAGE or PRAK constructs by using Lipofectamine LTX (Invitrogen) according to the manufacturer’s protocol. PRAK siRNA (three to five target-specific 19–25 nt siRNAs designed to knockdown gene expression) or scrambled cotrol siRNA (Santa Cruz Biotechnology) by using RNAiMAX (Invitrogen) according to the manufacturer’s protocol.

### Reagents

Aβ_42_ peptide (AP62-0-80; American Peptide;2 μM) was dissolved in hexafluoroisopropanol for 4 days at room temperature. The lyophilized peptide was then dissolved in DMSO (Sigma) [54]. For this experiments, an monomeric preparation of Aβ_42_ peptide was utilized, that was characterized by atomic force microscopy (AFM). Bafilomycin A_1_ (Sigma; 10nM) was dissolved in DMSO and pretreatment 1 h before Aβ treatment.

### Immunoprecipitation

CHO cells transfected with RAGE and GFP-tagged PRAK were washed with PBS and lysed with 1 % CHAPS buffer (Sigma). To reduce non-specific binding, pre-clearing with protein A/G agarose (Santa Cruz Biotechnology) was performed for 1 h at 4 °C with gentle rotating. After bicinchoninic acid assay, equal protein lysates were immunoprecipitated with anti-GFP antibodies (1 μg/mL; Santa Cruz Biotechnology), incubated overnight at 4 °C with gentle rotating, and added to the beads for 1 h. The samples were washed in the lysis buffer and elution protein complex with the SDS-PAGE sample loading buffer and analyzed by western blotting as described above. Brain tissue or SH-SY5Y cells were lysed with RIPA and using ImmunoCruz™ IP/WB Optima E System (Santa Cruz Biotechnology) according to the manufacturer’s protocol with anti-RAGE antibodies (1 μg/mL; Millipore).

### Western blot

Cells were washed with PBS and lysed in RIPA buffer supplemented with a proteinase and phosphatase inhibitor cocktail (Sigma). For whole cell lysates, cells were sonicated and centrifuged for 20 min at 17,950 g at 4 °C. Cell lysates were run on SDS-PAGE gels, and then transferred to PVDF membrane. After overnight incubation at 4 °C with the primary antibody in 3 % BSA, the signal was enhanced using Enhanced chemiluminescence (ECL, GE Healthcare Biosciences) followed by image analysis with Bioimaging analyzer (LAS-3000, Fuji Film, Inc.) and Multi-Gauge (Fuji). Primary antibodies were used against RAGE (Millipore), LC3B, p-ULK1 (S757), ULK1, p-mTOR (S2481), mTOR, p-p70s6k, p70s6k, p-p38, p38, p-IkB, IkB, tubulin (Cell Signaling Technology), PRAK, GFP (Santa Cruz Biotechnology), and actin (Sigma). Anti-p-PRAK (T182) and anti-p-Rheb (S130) antibodies were obtained from Jiahuai Han’s Lab (Xiamen University).

### Surface plasmon resonance spectroscopy (SPR)

The sensor chip CM5 with pre-immobilized anti-GST antibodies in one flow cell was first saturated with GST-fused PRAK protein. To analyze the binding kinetics, multiple concentrations of RAGE C-terminal region were diluted in HBS-EP buffer (0.01 M HEPES, pH 7.4; 0.15 M NaCl; 3 mM EDTA; 0.005 % Surfactant P20) and infused onto the sensor chip at a flow rate of 30 μL/min for 180 s. The response unit (RU) was recorded in real time by Biacore (Biacore X-100 plus, Biacore, Inc.). After the analyte infusion was stopped, the HBS-EP buffer was poured over the chip for 420 s at a flow rate of 30 μL/min. In order to dissociate the bounded analytes from the immobilized PRAK and for acquisition of the dissociation curves. Injection of 1 % PBS, including the HBS-EP buffer was performed as the vehicle control. The Biacore control software was used to measure the changes in plot, the binding curve, and RU. The curves obtained from the SPR experiments were analyzed and the dissociation equilibrium constant for RAGE C-terminus to immobilized PRAK was calculated using kinetic evaluation software. The dissociation constant KD (M) was derived from the equation, KD = kd/ka, where kd and ka are dissociation-and association-rate constants, respectively.

### In Situ Proximity Ligation Assay

SH-SY5Y cells were co-transfected with RAGE and PRAK, stimulated with 2 μM of Aβ for 6 h, and stained according to the manufacturer’s instructions (Duolink; 92008, Olink Bioscience).

### Immunofluorescence assay

For immunocytochemistry, cells were fixed with 4 % PFA for 20 min at RT. Incubation was performed with 5 % BSA in PBS containing 1 % Triton X-100 for 30 min for blocking and permeabilization. The cells were then incubated with primary antibodies against human RAGE (1:200; R&D Systems) and PRAK (1:500; Santa Cruz Biotechnology) at 4 °C overnight. Finally, the cells were incubated with anti-rabbit Alexa 488 and anti-goat Alexa 594 (Invitrogen) at RT for 1 h, followed by DAPI staining and rinsing. The fluorescence was visualized by a super resolution structured illumination microscopy (Nikon N-SIM, Nikon Instruments Inc.).

### Transmission electron microscopy (TEM)

Cells were fixed with 2 % PFA in 0.1 M phosphate or cacodylate buffer (pH 7.2) and 2.5 % glutaraldehyde in 0.1 M phosphate buffer (pH 7.2) at 4 °C for 24 h. Cells were then embedded with epoxy resin and polymerized at 38 °C for 12 h and then at 60 °C for 48 h. Thin sections, cut on an ultramicrotome (MT-XL, RMC Products), were collected on a copper grid. Samples were thin sectioned at 65 nm. Sections were then stained with 4 % lead citrate and saturated 4 % uranyl acetate, and examined under an electron microscope (JEM-1400, JEOL) at 80 kV [55]. Counting the number of autophagic structure per sheet (n = 6), using photographs taken at 20,000x magnification.

### Statistical analysis

For statistical analysis, the unpaired *t*-test or the One-Way/Two-Way ANOVA was performed using Graphpad Instat 5.1 (GraphPad Software Inc.). Data in figures represent mean ± SEM.

## Additional files

Additional file 1: Figure S1. Interaction between endogenous PRAK and RAGE. SH-SY5Y cell lysates were immunoprecipitated with RAGE antibody or normal IgG antibody. Western blot analysis performed with the indicated antibodies. (TIFF 308 kb) Additional file 2: Figure S2. PRAK does not mediate NF-κB activation through Aβ-RAGE interaction. SH-SY5Y cells were transfected with plasmids expressing RAGE and then transiently re-transfected with PRAK siRNA or scrambled control siRNA. After 24 h, cells were treated with DMSO or 2 μM monomeric Aβ for 30 min and western blot analysis performed with the indicated antibodies. (TIFF 532 kb) Additional file 3: Figure S3. RAGE-independent pathway activates p38 via Aβ. RAGE and DN-RAGE-overexpressing SH-SY5Y cells were treated with either DMSO or 2 μM monomeric Aβ for 6 h. Western blot analysis performed with the indicated antibodies. (TIFF 494 kb)

## Abbreviations

AD: Alzheimer’s disease
Aβ: Amyloid beta
DN-RAGE: dominant negative RAGE
GFP: green fluorescent protein
IP: immunoprecipitation
LC3: microtubule-associated protein 1 light chain 3
mTORC1: mammalian target of rapamycin complex 1
NF-κB: nuclear factor of kappa light polypeptide gene enhancer in B-cells
PRAK: p38-regulated/activated protein kinase
RAGE: receptor for advanced glycation endproducts
Rheb: ras homologue enriched in brain
SPR: surface plasmon resonance
TEM: transmission electron microscopy
ULK1: UNC-51-like kinase 1
