# Supplementary figures and images for: The novel RAGE interactor PRAK is associated with autophagy signaling in Alzheimer’s disease pathogenesis
Source: Mol Neurodegener. 2016 Jan 12;11:4. doi: 10.1186/s13024-016-0068-5 (PMC4709948; doi:10.1186/s13024-016-0068-5)

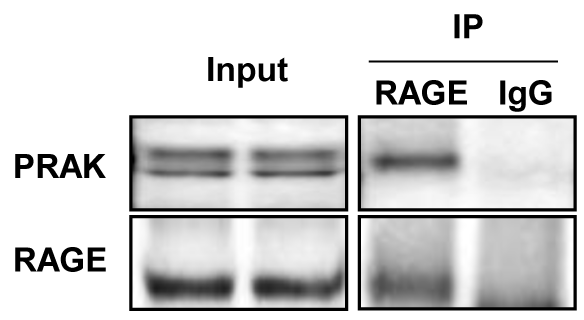

Supplement: Additional file 1: Figure S1. — Interaction between endogenous PRAK and RAGE. SH-SY5Y cell lysates were immunoprecipitated with RAGE antibody or normal IgG antibody. Western blot analysis performed with the indicated antibodies. (TIFF 308 kb) [file 13024_2016_68_MOESM1_ESM.tiff]

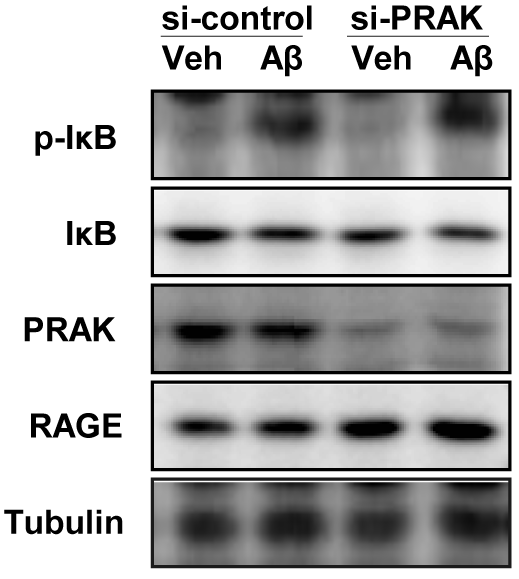

Supplement: Additional file 2: Figure S2. — PRAK does not mediate NF-κB activation through Aβ-RAGE interaction. SH-SY5Y cells were transfected with plasmids expressing RAGE and then transiently re-transfected with PRAK siRNA or scrambled control siRNA. After 24 h, cells were treated with DMSO or 2 μM monomeric Aβ for 30 min and western blot analysis performed with the indicated antibodies. (TIFF 532 kb) [file 13024_2016_68_MOESM2_ESM.tiff]

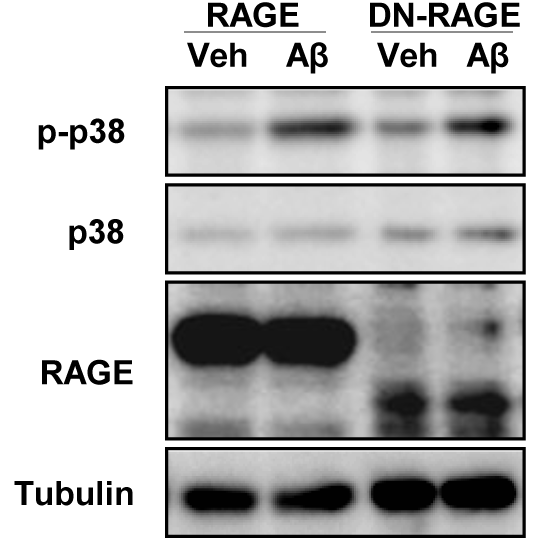

Supplement: Additional file 3: Figure S3. — RAGE-independent pathway activates p38 via Aβ. RAGE and DN-RAGE-overexpressing SH-SY5Y cells were treated with either DMSO or 2 μM monomeric Aβ for 6 h. Western blot analysis performed with the indicated antibodies. (TIFF 494 kb) [file 13024_2016_68_MOESM3_ESM.tiff]
